# Is Neoadjuvancy with Imatinib Useful Before Mohs Surgery in Locally Advanced Dermatofibrosarcoma Protuberans? Experience of a Dermato-Oncology Referral Center

**DOI:** 10.3390/ijms27073204

**Published:** 2026-04-01

**Authors:** A. Bota-Llorca, B. Llombart-Cussac, C. Serra-Guillén, J. A. López-Guerrero, V. Traves, J. J. Sanmartín-Martínez, C. Requena, E. Nagore, J. Padilla-Esquivel, D. Millán-Esteban, F. Mayo, O. Sanmartín

**Affiliations:** 1 Doctoral School, Catholic University of Valencia San Vicente Mártir, 46001 Valencia, Spain; anna.bota.llorca@gmail.com (A.B.-L.); cserraguillen@gmail.com (C.S.-G.); jalopez@fivo.org (J.A.L.-G.); enagore@fivo.org (E.N.); osanmartinj@gmail.com (O.S.); 2 Dermatology Department, Fundación Instituto Valenciano de Oncología, 46009 Valencia, Spain; jose.j.sanmartin@uv.es (J.J.S.-M.); celiareq@hotmail.com (C.R.); david.millan@ucv.es (D.M.-E.); mayomartinezf@gmail.com (F.M.); 3 Laboratory of Molecular Biology, Fundación Instituto Valenciano de Oncología, 46009 Valencia, Spain; 4 Pathology Department, Fundación Instituto Valenciano de Oncología, 46009 Valencia, Spain; vtraves@fivo.org (V.T.); jpadillaesquivel@gmail.com (J.P.-E.)

**Keywords:** dermatofibrosarcoma protuberans, locally advanced, imatinib, tyrosine kinase inhibitors, Mohs surgery, modified Mohs surgery

## Abstract

Dermatofibrosarcoma protuberans (DFSP) is a rare cutaneous sarcoma in which complete surgical excision is standard treatment, although some tumors are initially unresectable. Neoadjuvant imatinib has been proposed in these cases, but data on its histopathological and molecular effects and long-term outcomes remain limited. To evaluate the clinical, histopathological and molecular impacts of neoadjuvant imatinib prior to modified Mohs surgery (MMS) in locally advanced DFSP. Single-center, retrospective study. After a mean of 10 months of neoadjuvant imatinib, partial tumor size reduction was observed in 60% of patients (mean reduction 37.8%), while the remaining cases showed disease stabilization; no complete responses were recorded. All tumors exhibited marked volumetric and consistency reduction, with histology revealing extensive hypocellular hyaline regression and attenuated CD34 and nestin expression. Persistence of COL1A1–PDGFB fusion transcripts was detected in post-treatment samples. Following MMS, local recurrence occurred in 30% of patients at long-term after a mean of 10.8-year follow-up since the last surgery. Neoadjuvant imatinib in locally advanced DFSP results in tumor volume reduction without decreasing the final surgical defect. The histological response is typically patchy and may compromise detection of residual disease, potentially increasing the risk of local recurrence.

## 1. Introduction

Dermatofibrosarcoma protuberans (DFSP) is a rare, slow-growing cutaneous sarcoma that accounts for 0.1% of total malignancies. It usually appears between the second and fifth decades of life and arises most commonly in the trunk and proximal part of the extremities [1]. Although head and neck DFSP represents only 10–15% of cases, these tumors often require complex surgical management due to the difficulty of achieving wide excisions without significant cosmetic or functional impairment, which contributes to higher local recurrence rates and an increased risk of distant metastasis [2].

Histologically, DFSP is characterized by a dermal proliferation of monomorphic spindle cells arranged in a storiform or cartwheel pattern, with diffuse positivity to CD34 immunostaining [3]. Tumor cells frequently extend into the subcutaneous tissue through asymmetric, finger-like projections that can reach significant distances from the center of the tumor and can infiltrate deeper structures such as muscle or periosteum, often leading to incomplete resection and high recurrence rates. Positivity to nestin immunostaining is ubiquitous in DFSP (98.6–100%) [4], and high expression levels have been linked to longstanding, deeper lesions and a poorer prognosis [5].

DFSP typically involves a chromosomal translocation t(17;22) (q22;q13), which results in the formation of a fusion gene involving the Collagen Type 1 Alpha 1 (*COL1A1*) gene and the Platelet Derived Growth Factor Beta (*PDGFB*) gene. This fusion generates a chimeric functional protein that causes a constant activation of the PDGFβ receptor (PDGFRB), a tyrosine kinase that acts as a potent growth factor and induces tumor cell proliferation through an autocrine signaling loop [6]. The *COL1A1-PDGFB* fusion transcript can be detected by reverse transcriptase-polymerase chain reaction (RT-PCR) or fluorescence in situ hybridization (FISH) in up to 96% of DFSP cases [7,8]. In recent years, RT-PCR-based studies have led to the identification of novel fusion genes in patients who are negative for the canonical COL1A1–PDGFB fusion. Among these, fusions involving COL1A2–PDGFB have been described, as well as rearrangements affecting PDGFD [7].

Complete surgical excision with negative histologic margins remains the gold standard treatment for DFSP, preferably using margin-controlled techniques, given the tumor’s infiltrative and asymmetric growth pattern [9,10]. Given that DFSP tends to present as large, ill-defined lesions, margin-controlled excision techniques using paraffin-embedded sections (slow Mohs or modified Mohs surgery, MMS) are generally favored over frozen sections due to their superior histological quality [11,12]. However, some cases of DFSP may be considered locally advanced and may not be initially suitable for surgical treatment, particularly when located in cosmetically or functionally sensitive areas such as the head and neck. In these cases, provided that the *COL1A1-PDGFB* fusion is present, systemic treatment with tyrosine kinase inhibitor, imatinib, has been proposed as neoadjuvant therapy to reduce tumor burden and facilitate subsequent surgical resection. Previous studies have reported encouraging results using neoadjuvant imatinib, with up to 60.5% of patients experiencing partial or complete tumor response [13]. However, most series include short-term follow-up or lack histological confirmation of tumor clearance [14,15], and evidence about long-term efficacy after imatinib followed by MMS remains limited.

The aim of this study was to evaluate the clinical, histopathological and molecular effects of imatinib as a neoadjuvant treatment in locally advanced DFSP and assess the long-term outcomes following salvage surgery by MMS.

## 2. Results

### 2.1. Patient Demographics

Our study included ten patients treated with imatinib, six women and four men, with a mean age of 40.6 years. The mean interval from patient tumor detection to treatment initiation was 16.25 years. Patient general characteristics are shown in Table 1. Most tumors (6/10, 60%) appeared on the face or scalp, and the rest were located in the neck or upper thorax. All of them infiltrated deep structures like muscle or periosteum. None of the tumors invaded the bone.

Mean tumor size was 7.5 × 5.4 cm on clinical evaluation. Comparison between clinical examination and MRI measurements before and after treatment with imatinib can be seen in Table 1. Tumor size assessment showed substantial discrepancies between clinical examination and MRI in 8/10 (80%) cases, with imaging studies generally underestimating the true tumor extent. Regarding depth of infiltration, concordance between MRI findings and histopathological analysis was observed in only 3/10 (30%) cases.

### 2.2. Treatment Outcomes

**Figure 1:**
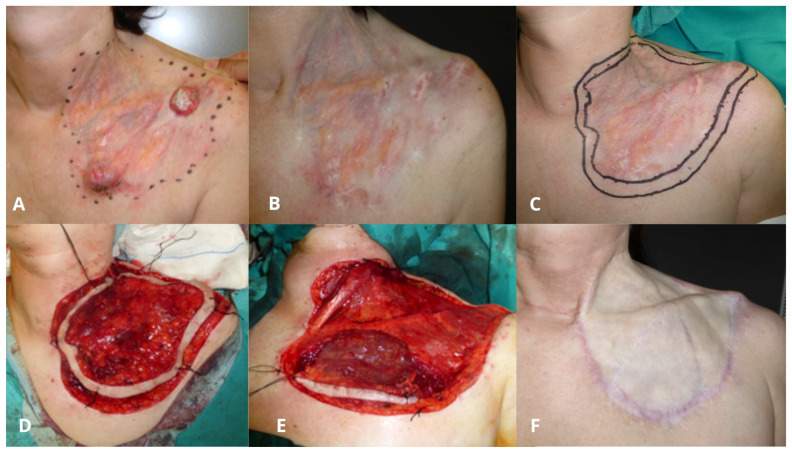
Clavicular DFSP considered unresectable due to extensive surface involvement and proximity to cervical vascular structures. (**A**) Initial lesion measuring 15 × 11.5 cm. (**B**) Appearance after 4 months of imatinib therapy, showing a marked reduction in tumor nodule volume without an apparent decrease in overall diameter. (**C**) Clinically palpable tumor and planned excision. (**D**) First stage of MMS, including the muscular fascia. (**E**) Second stage of MMS due to infiltration of the pectoralis muscle. (**F**) Final defect of 17 × 17 cm reconstructed with a full-thickness skin graft to facilitate follow-up.

**Figure 2:**
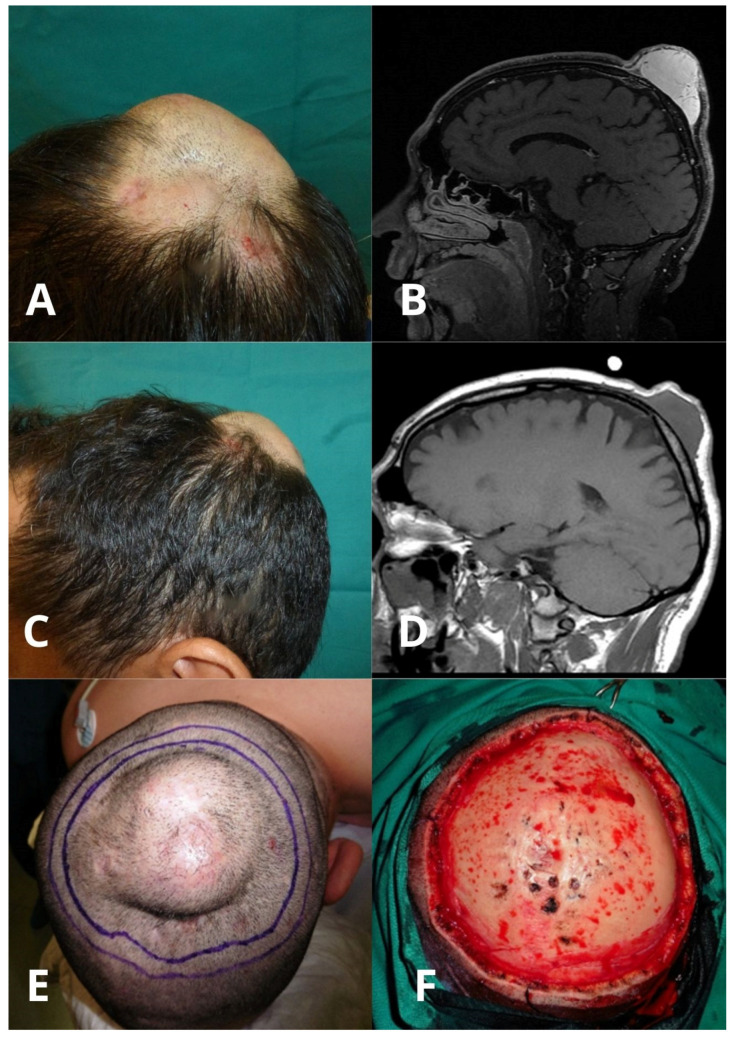
Extensive DFSP on the scalp. Clinical (**A**) and MRI (**B**) before imatinib, and clinical (**C**) and MRI (**D**) imaging after 3 months of imatinib therapy, with visible tumor volume reduction. (**E**) Surgical planning for the first MMS stage. (**F**) Final surgical defect with signs of tumor compression of the calvaria.

Progressive reduction of the tumor was sustained during a mean of 5 months (range 2–9) after starting imatinib, and the treatment was maintained for a mean of 10 months (range 6–12) before surgery. A mean tumor size reduction of 37.8% (range 13.3–91.3%) was observed in 6/10 (60%) of patients. In addition, 4/10 patients (40%) showed stabilization with no clinical size modification. None of the tumors experienced a complete response. All cases showed a remarkable reduction in tumor volume and consistency on palpation, even when the diameter was not decreased [Figure 1 and Figure 2].

Imatinib therapy was generally well tolerated, with 7/10 (70%) patients reporting G1-2 adverse effects, most frequently nausea and facial edema. No grade 3 or 4 adverse effects were reported. Only 1/10 (10%) patient required a short treatment discontinuation due to G2 nausea and asthenia, which was later resolved on reintroduction.

Surgical treatment was performed after a mean of 10 months after starting imatinib. Most patients (7/10, 70%) experienced tumor clearance after two MMS stages, 2/10 (20%) required three stages and 1/10 (10%) required six stages (mean 2.6 stages). Mean post-surgical defect size was 9.85 × 7.75 cm. The results can be seen in Table 1.

### 2.3. Changes in Histology and Molecular Biology

**Figure 3:**
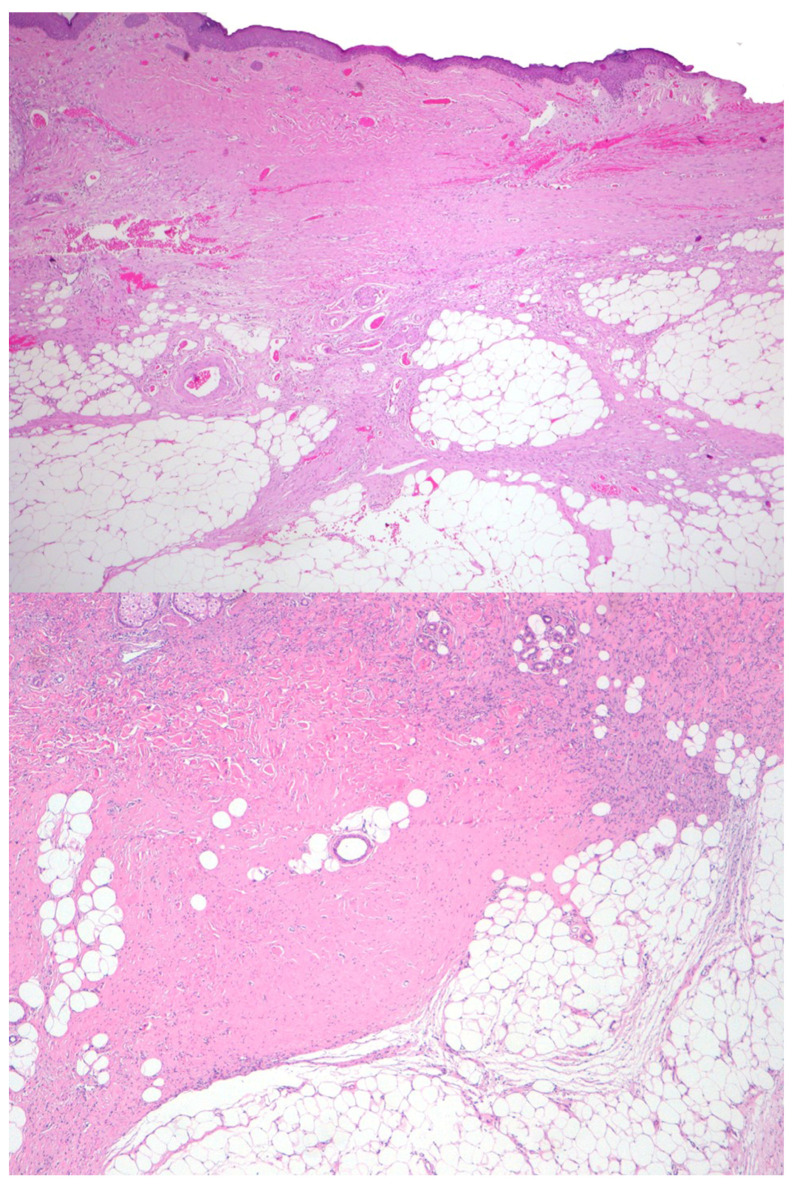
Histologic alterations after imatinib therapy. (Top) Low-power view (×40) showing marked reduction in tumor cellularity in the dermis; (Bottom) Higher magnification (×100) showsmore cellular foci persist, composed of spindle-shaped tumor cells infiltrating the subcutaneous tissue through the septa in a digitiform pattern.

**Figure 4:**
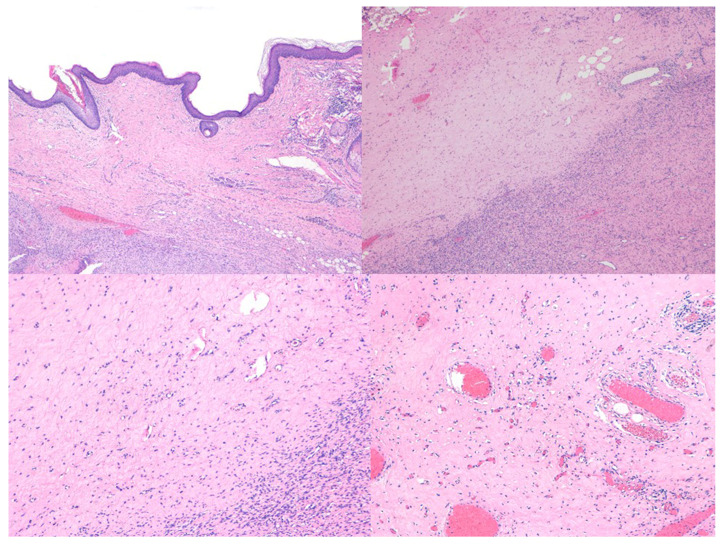
Histological changes in Dermatofibrosarcoma protuberans after treatment with imatinib (H&E stain). (Top left) Low-power vies (×40) showing extensive areas of hypocellularity with dense hyalinized stroma alternating with residual more cellular regions. (Top right, Bottom left, Bottom right) Higher magnification (×100) demonstrating sparse spindle cells embedded in a collagenized stroma, alongside focal areas with increased cellularity composed of uniform spindle-shaped tumor cells.

Histological evaluation revealed the presence of hypocellular hyaline regression, characterized by markedly reduced cellularity and replacement of tumor cells by dense hyalinized stroma [Figure 3]. Hypocellular areas were patchily distributed, comprising 50–90% of the total tumor area, and alternated with hypercellular foci showing dense proliferation of spindle-shaped neoplastic cells [Figure 4]. Additional histological patterns, including myxoid degeneration and thick collagen bundles, were also observed in some cases.

All tumors initially expressed moderate-to-high intensity CD34 and nestin immunostaining. After imatinib, all cases showed positive but attenuated CD34 immunostaining in tumoral cells. In addition, there was a complete loss of nestin expression in 8/10 (80%) cases and a marked reduction in 2/10 (20%).

Post-imatinib FISH analysis performed in the tumor debulking tissue yielded positive results in 6/10 (60%) cases [Table 2].

### 2.4. Long-Term Outcome

In total, 3/10 (30%) patients showed local relapses after MMS, with two of them on two occasions. Recurrences appeared remarkably late in the follow-up, at 55, 79 and 155 months. Tumors were again treated with MMS, requiring two to eight MMS stages to obtain tumor clearance. All patients remain free of disease after a mean of 129.7 months since the last surgery. No metastases were detected in the follow-up.

## 3. Discussion

This study evaluates the role of imatinib at a clinical, histopathological and molecular level as a neoadjuvant therapy prior to surgical excision in locally advanced DFSP in the longest follow-up case series to date. However, a higher recurrence rate was observed even after performing MMS, and relapses appeared late in the follow-up. None of the tumors of our series experienced a complete histologic response after imatinib alone.

Imatinib is a selective tyrosine kinase inhibitor targeting BCR-ABL, KIT and PDGFR α and β, and has been approved by the European Medicines Agency (EMA) since 2011 for the treatment of locally advanced or metastatic DFSP [16]. Encouraging response rates reported in the literature have led to the consideration of this agent as an off-label neoadjuvant approach before surgery. Previous studies have reported promising results, with partial therapeutic responses observed in 70% of cases [17,18] and patients experiencing a mean tumor size reduction of 31.5–36.9% [15,19]. Our results are consistent with these findings. As in gastrointestinal tumors (GIST), imatinib is not curative in DFSP [18,20], but it may be used to reduce tumor burden in selected patients with inoperable tumors or metastatic disease [17].

DFSP treated with imatinib usually expresses a dramatic decrease in tumor volume and obvious histologic changes, with marked reduction in cellularity and mitotic rate, and tumoral tissue is exchanged with abundant amounts of hyalinized collagen and no necrosis [13,21,22]. These alterations are found even when there is no clinical tumor size reduction and can be induced in a diffuse or patchy fashion [23], which creates discontinuous tumor areas that can hinder the detection of the true negative margin in surgical samples, even with margin-controlled techniques. In addition, CD34 immunostaining can be attenuated after imatinib, which may make the detection of sparse tumoral cells scattered in fibrous areas in the periphery of the tumor difficult. In our series, CD34 immunostaining remained positive in tumoral areas but was diminished or negative in hyalinized areas. Nestin immunostaining has been associated with local aggressiveness in several tumors, including large, long-standing DFSP with deep infiltration [5]. In this series, nestin was initially strongly positive in all tumors, likely reflecting the long-standing nature of the lesions and their extension into deep anatomical structures. However, nestin staining turned negative in almost every case after imatinib, probably due to the dramatic reduction of tumor burden. Nestin immunostaining has not been previously evaluated in this therapeutic context, and, therefore, no comparative data are available to assess its utility. In our study, the absence of nestin expression did not provide information on the persistence of residual tumor during MMS evaluation. Further studies are needed to clarify the potential role of nestin in this setting.

Interestingly, in surgical samples, where histological interpretation is challenging, the *COL1A1-PDGFB* fusion was detectable in most cases by FISH analysis performed on debulking tissue obtained during MMS. These findings are in line with the previous study by Stacchiotti et al. [18] and indicate that imatinib treatment does not result in loss of *COL1A1–PDGFB* expression, neither in the tumor nor in areas of hyaline regression. In the present study, molecular analyses were performed at two different time points: RT-PCR was carried out before imatinib treatment, whereas FISH was performed on tumor debulking specimens during MMS after imatinib, preventing direct comparison between the two techniques. However, previous reports have suggested that FISH may be more sensitive and easier to perform in paraffin sections than RT-PCR, supporting its use as the preferred diagnostic approach [1,24]. More studies should be carried out in order to establish the utility of molecular biology techniques during MMS.

Whether the presence of fusion gene *COL1A1-PDGFB* predicts response to imatinib remains controversial. Most studies and guidelines recommend routine confirmation of the fusion gene before starting tyrosine kinase inhibitors, but its presence does not predict histologic response [15]. Clinical response is usually visible after the first 2–3 months of treatment and generally peaks after 5 to 6 months [25]. Primary and secondary resistance to imatinib has been described, possibly associated with low levels of phosphorylation of PDGFRB [15].

Current guidelines suggest using imatinib as neoadjuvant therapy in selected cases, always after a multidisciplinary consensus is reached. In the NCCN 2025 update, imatinib is advised in patients with persistently unresectable DFSP following repeated resections, or when additional surgery is expected to entail significant functional or cosmetic impairment [26]. European 2024 guidelines suggest that imatinib may be a potential neoadjuvant treatment in difficult-to-resect or unresectable DFSP [27], but they address the possibility of dedifferentiation after treatment, which could alter the reliability of margin assessment. Although all the tumors in our series were amenable to salvage surgery after imatinib therapy, treatment-induced histologic modifications may have hindered the detection of minimal residual tumor during MMS evaluation. We observed an unexpectedly high local recurrence rate, with two patients developing multiple recurrences during follow-up. In contrast, our previously reported recurrence rate in DFSP after MMS was 0.9% [28], in line with the previous literature [9]. This discrepancy is likely attributable to the patchy histologic response to imatinib, which generates discontinuous foci of residual tumor and subclinical extension. In addition, all tumors in this series were located in complex regions, such as the head and neck and upper thorax, which are known to require more complex surgeries and more MMS stages due to their subcutaneous nature and subclinical extension through deep structures [29,30], and therefore already have a higher risk of local recurrence. The tumors in this series required a mean of 2.6 MMS stages to obtain clear margins, compared to a mean of 1.47 MMS stages in our largest published cohort of 222 DFSP [28]. Interestingly, in our series, neoadjuvant imatinib did not result in a reduction of the final surgical defect, suggesting that some tumors initially classified as unresectable or surgically challenging might, in fact, have been amenable to primary surgical management.

Overall, imatinib therapy is well tolerated with mild adverse effects, and only 5% of patients require treatment discontinuation due to complications [31]. Better tolerance to the treatment can be managed with the lower dose of 400 mg daily, which exhibits a similar response rate to higher doses [13,18,32]. In our study, we homogeneously administered 400 mg daily, even if patients showed no tumor size reduction, and no G3 or G4 adverse events associated with imatinib were reported.

Alternative systemic therapies beyond imatinib in DFSP are supported by limited and heterogeneous evidence. Antiangiogenic agents such as apatinib have only been described in isolated case reports, with partial responses in metastatic settings and short follow-up [33]. Sunitinib has shown moderate activity in retrospective series after imatinib failure, with a response rate around 40%, and with manageable but relevant toxicity [34]. In contrast, pazopanib, evaluated in a phase II trial, demonstrated lower response rates (22–30%) and a substantial toxicity burden, leading to treatment discontinuation in 39% of patients, with a short median follow-up of 6.2 months [35]. Overall, despite occasional clinical benefit, these alternative agents show lower efficacy and a less favorable safety profile compared with imatinib, and the evidence is largely based on small cohorts or early-phase studies. Therefore, their use should be restricted to highly selected cases without surgical options after multidisciplinary evaluation by experienced teams, or in the metastatic setting with a palliative intent. Pembrolizumab has also been proposed as a potential alternative treatment for unresectable dermatofibrosarcoma protuberans; however, recent studies have shown an absence of microsatellite instability in these tumors, a common pathway associated with response to immunotherapy, highlighting the need for further research exploring alternative predictive mechanisms [36].

The use of imaging techniques can provide helpful information for preoperative planning and follow-up of cutaneous sarcomas. MRI is the imaging modality of choice for the evaluation of head and neck DFSP, which typically appears as a T2-hyperintense lesion with marked contrast enhancement [37]. MRI has proven utility in estimating the depth of tumor invasion, showing good histological correlation in superficial lesions extending to the subcutis. However, its accuracy decreases in tumors located in the head, neck and upper thorax, both in tumor size delimitation and in the assessment of depth infiltration [38]. In our series, tumor size and depth of infiltration were frequently discordant between clinical examination, MRI and histopathological findings. The complex regional anatomy of the head and neck, together with the thin anatomical structures, prior surgical scarring and treatment-related changes induced by imatinib, likely contributed to altered MRI signal characteristics and limited accuracy in tumor delineation. Consequently, imaging alone cannot reliably assess residual disease after imatinib or surgery, and tumor clearance should always be confirmed histopathologically. Nevertheless, MRI remains valuable for initial assessment, preoperative planning [39] and follow-up, particularly for the early detection of local recurrence [40].

This study presents several limitations, mainly its retrospective nature and the reduced number of cases, which prevent statistical analysis. In addition, the duration of treatment with imatinib was heterogeneous and depended on the dermatologist and oncologist’s criteria. Nevertheless, the dosage was homogeneous. Finally, the included cases were initially complex and located in cosmetically sensitive areas, which may have led to an overestimation of recurrence rates. The main strength of this study is the long-term follow-up, which has allowed detecting late recurrences. Generally, DFSP relapses appear more frequently in the first 5 years after surgery, but later recurrences have been reported. The lower residual cellularity rate after imatinib may delay the clinical detection of recurrences. Most studies describe shorter follow-ups, usually up to 5 years [19,23], which can overestimate the long-term efficacy of systemic treatments.

## 4. Materials and Methods

### 4.1. Study Design

We designed a retrospective, single-center, observational study including patients with histologically confirmed DFSP, treated with imatinib prior to MMS between 2006 and 2025 at the Fundación Instituto Valenciano de Oncología (FIVO) in Valencia, Spain. We collected data from patients with primary or recurrent DFSP that were initially considered highly complex, with a high risk of aesthetic disfigurement, and therefore selected by a multidisciplinary tumor board for neoadjuvant treatment with imatinib in order to reduce tumor size and facilitate subsequent surgery. The board included dermatologists, ear–nose–throat surgeons, plastic surgeons, medical and radiation oncologists and radiologists. Metastatic tumors were excluded. Tumors were selected based on tumor location, tumor size or the inability to achieve clear surgical margins.

This project was approved by the local institutional research ethics committee. Written informed consent was obtained from all participants.

### 4.2. Tumor Characteristics

Demographic data was collected from the clinical database at the FIVO Department of Dermatology. Tumor size was registered in centimeters (cm) based on both clinical and radiological assessments of the major and minor diameters. Clinical measurement of tumor size was used for follow-up.

Histological examination of all available hematoxylin and eosin-stained slides was performed before and after imatinib treatment by a trained pathologist and two dermatologists (V. T., A. B., and B. L.). All histologic samples were immunostained using the commercially available nestin mouse monoclonal IgG1 antibody (10c2) (Santa Cruz Biotechnology, Inc., Dallas, TX, USA) and CD 34 mouse monoclonal IgG1 antibody (QBEnd 10) (Dako Cytomation, Copenhagen, Denmark), both at a 1:50 dilution. Immunostaining was classified in the following categories according to the percentage of positive tumor cells (-, <5%): low (+, 5–30%), intermediate (++, 30–60%), or high (+++, >60%).

The *COL1A1-PDGFB* translocation was confirmed in all patients by molecular testing at two time points: before and after completion of imatinib therapy. Molecular analyses were performed using RT-PCR and FISH on formalin-fixed paraffin-embedded (FFPE) tumor sections, as described elsewhere [41]. Post-imatinib FISH was carried out on histological sections obtained from Mohs surgery debulking tissue.

### 4.3. Systemic and Surgical Treatment

Patients received neoadjuvant imatinib mesylate 400 mg (Glivec^®^, Novartis-Pharma, Basel, Switzerland) once daily. The lower dosage of imatinib was chosen due to previous studies reporting similar therapeutic results with better tolerance and fewer adverse events [6,13]. Therapy duration was not initially defined and ranged from six to twelve months upon the dermatologist and oncologist’s assessment. Treatment was discontinued when no further reduction in tumor size and/or induration was observed despite continuation of therapy for at least two months.

Baseline evaluation included physical examination, blood tests and magnetic resonance imaging (MRI). During treatment, patients were evaluated with physical examination and blood tests every month for the first three months, and every two months thereafter until surgical excision. MRI was performed every three to six months until surgery. All patients were treated surgically using the Modified Mohs Surgery (MMS) technique used in our department [28]. The visible tumor (or the scar of a previously treated tumor) was debulked by excision, and the first Mohs layer was taken with a 1 cm tissue margin around and under the wound. The tissue specimen was oriented with silk sutures, photographed, sequentially divided into multiple specimens, mapped for precise anatomic orientation, and sent to the pathology technician for formalin fixation and paraffin embedding, before undergoing horizontal sectioning. Sections were stained with hematoxylin and eosin, and diagnosis was confirmed by CD34 immunostaining. Results were reported to the dermatologists within 48 h. In patients with positive margins, an additional 0.5 to 1 cm layer was excised according to the anatomic map. The cyclic process of excision, mapping, and microscopic examination was repeated until no tumor was microscopically detected.

Postoperative follow-up consisted of physical examination at least every six months during the first five years and annually afterwards, with annual MRI.

## 5. Conclusions

Our findings suggest that the role of imatinib as neoadjuvant therapy prior to surgery is limited. Although imatinib reduces tumor cellularity and volume, it substantially impairs margin assessment and may thus increase the risk of local recurrence. Extended follow-up beyond the recommended 5 years appears warranted in these patients. Moreover, neoadjuvant imatinib did not reduce the final surgical defect, suggesting that some tumors initially considered locally advanced may have been suitable for upfront surgery. Accordingly, imatinib should be reserved for metastatic disease or carefully selected unresectable cases following multidisciplinary evaluation.

## Figures and Tables

**Table 1:** Summary of general data, including the clinical, histological, radiological, and therapeutic characteristics of the patients included in the study.

Patient ID	1	2	3	4	5	6	7	8	9	10
Sex	M	F	M	F	M	F	F	M	F	F
Age	33	42	39	47	25	41	43	39	50	47
Location	Cheek	Shoulder	Scalp	Clavicle	Lower eyelid	Shoulder	Clavicle	Cheek	Forehead	Scalp
Time to diagnosis ^a^ (years)	23	10	1	47	0.5	20	18	11	2	30
Tumor type	Primary	Recurrent	Primary	Primary	Primary	Primary	Recurrent	Primary	Primary	Recurrent
Initial clinical size (cm)	6.5 × 4	6 × 5	12 × 12	15 × 11.5	3.5 × 2	9 × 3	4.5 × 3	6 × 4	4.3	8.5 × 6.5
Initial MRI size (cm)	(ill-defined)	5 × 4	7.8 × 6	3.5 × 3	3.3 × 1.7	7.8 × 3.9	2.6 × 1.8	1.7 × 1.5	3.5 × 1.8	5.7 (ill-defined)
Final clinical size (cm)	6.5 × 4	6 × 5	5 × 2.5	13 × 11	2.5 × 1.5	9 × 3	3 × 0.5	5 × 3	4 × 3	8.5 × 6.5
Final MRI size (cm)	2 × 1.2	(ill defined)	6 × 5.8	(ill defined)	2.2 × 1.3	7.8 × 3.9	1.2 × 0.7	0.9 × 0.6	1.9 × 1	5.7 (ill-defined)
Final defect after MMS (cm)	9 × 7	10 × 8	14 × 14	17 × 17	4.5 × 3	12 × 7	8 × 4.5	8 × 6	4 × 3	12 × 8
Histological depth of infiltration	Muscle	Periosteum	Muscle	Muscle	Muscle	Muscle	Muscle	Muscle	Periosteum	Periosteum
MRI depth of infiltration	Subcutis	Fascia	Periosteum	Periosteum	Subcutis	Fascia	Subcutis	Muscle	Periosteum	Periosteum
COL1A1-PDGFB (RT-PCR) ^b^	18	46	Non evaluable (FISH positive)	28	28	46	32	25	25	11
Treatment duration (months)	12	12	6	10	12	12	8	12	9	7
Tumor size reduction (%) ^c^	0	0	91.3	13.3	46.4	62.5	88.9	37.5	0	0
Therapeutic response interval time (months) ^d^	3	6	3	8	6	9	5	4	4	2
Adverse events related to imatinib	No	Nausea Eyelid edema (G1)	Nausea (G1)	Eyelid edema (G1)	No	No	Nausea Eyelid edema (G1)	Asthenia Nausea Diarrhea Facial edema (G2)	Asthenia Facial edema (G1)	Facial edema Diarrhea Xerosis Pruritus (G2)
MMS stages after imatinib	6	2	2	2	3	2	2	3	2	2
Time until recurrence after imatinib (months)	155	22 and 55	-	-	-	-	-	-	65 and 79	-
MMS stages after recurrence	8	3 and 2	-	-	-	-	-	-	3 and 2	-
Follow-up after last surgery (months)	83	158	83	136	157	104	176	145	139	116

^a^ Time from the patient’s detection of the lesion until the diagnosis of DFSP was made. ^b^ RT-PCR was routinely performed for the initial diagnosis of DFSP. COL1A1 exons are specified for each case, while the PDGFB breakpoint was located in exon 2 in all patients. ^c^ Tumor size reduction was calculated as follows: [(XY_is_ − XY_fs_)/XY_is_] × 100 (X: large diameter; Y: short diameter; is: initial size; and fs: final size). ^d^ Interval in which tumor size reduction was observed on clinical examination. MMS: Modified Mohs surgery; MRI: Magnetic Resonance Imaging.

**Table 2:** Histological, immunohistochemical and molecular biology evaluation before and after imatinib treatment.

ID	Previous CD34	Previous Nestin	Histology After Imatinib	Persistent Tumor Area (%)	Post Imatinib CD34 in Tumoral Areas	Post Imatinib CD34 in Hyaline Areas	Post Imatinib Nestin in Tumoral Areas	Post Imatinib Nestin in Hyaline Areas	Post Imatinib Fusion by FISH (Debulking)
1	+++	+++	Hypocellular hyaline regression	20	++	-	++	-	Positive
2	+++	+++	Hyaline regression with myxoid areas	50	+++	+	-	-	Positive
3	+++	+++	Hypocellular hyaline regression	50	+++	+	-	-	Non-evaluable
4	++	+++	Hyaline regression with thick collagen bundles	20	+	-	-	-	Non-evaluable
5	++	+++	Hypocellular hyaline regression	30	+	+	-	-	Positive
6	+++	+++	Thick collagen bundles	50	++	+	-	-	Positive
7	+++	+++	Hypocellular hyaline regression	10	+	-	+	-	Positive
8	+++	++	Hypocellular hyaline regression	50	+	+	-	-	Positive
9	+++	+++	Myxoid areas	15	+++	+	-	-	Non-evaluable
10	+++	+++	Hypocellular hyaline regression	10	++	-	-	-	Non-evaluable

## Data Availability

The original contributions presented in this study are included in the article. Further inquiries can be directed to the corresponding author.

## Abbreviations

The following abbreviations are used in this manuscript:

COL1A1	Collagen type 1 alpha 1
DFSP	Dermatofibrosarcoma Protuberans
FFPE	Formalin-fixed paraffin-embedded
FISH	Fluorescence in situ hybridization
MMS	Modified Mohs surgery
MRI	Magnetic resonance imaging
PDGFB	Platelet-derived growth factor beta
PDGFD	Platelet-derived growth factor beta
PDGFRB	Platelet-derived growth factor beta receptor
RT-PCR	Reverse transcriptase-polymerase chain reaction
